# Supplementary material for: Merkel Cell Polyomavirus in the Context of Oral Squamous Cell Carcinoma and Oral Potentially Malignant Disorders
Source: Biomedicines. 2024 Mar 22;12(4):709. doi: 10.3390/biomedicines12040709 (PMC11047982; doi:10.3390/biomedicines12040709)
Supplement: Supplementary file 1 [file biomedicines-12-00709-s001.zip › biomedicines-2914493-supplementary.pdf]

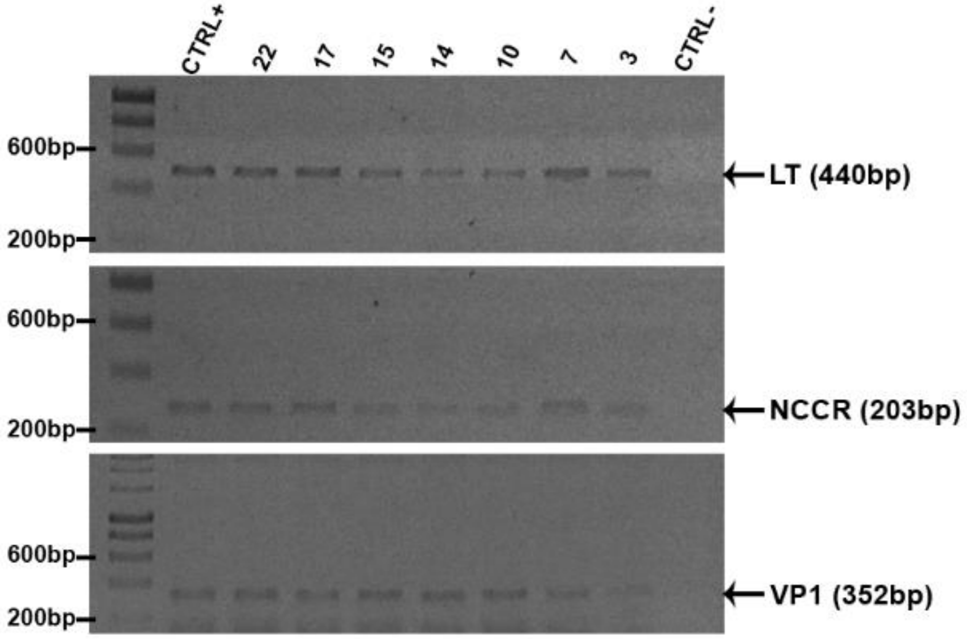

Figure S1. Representative results for the detection of MCPyV genome by PCR. The LT1, NCCR and VP1 primers produced amplicons of 440, 203 and 352 bp. CTRL+: positive control, CTRL-: water as the negative control. Molecular weights are indicated on the left.
